# The performance of pre-delivery serum concentrations of angiogenic factors in predicting postpartum antihypertensive drug therapy following abdominal delivery in severe preeclampsia and normotensive pregnancy

**DOI:** 10.1371/journal.pone.0215807

**Published:** 2019-04-25

**Authors:** Nnabuike Chibuoke Ngene, Jagidesa Moodley, Thajasvarie Naicker

**Affiliations:** 1 Department of Obstetrics and Gynaecology, University of KwaZulu-Natal, South Africa; 2 Women’s Health and HIV Research Group, Department of Obstetrics and Gynaecology, University of KwaZulu-Natal, South Africa; 3 Department of Optics and Imaging, University of KwaZulu-Natal, South Africa; Stellenbosch University, SOUTH AFRICA

## Abstract

**Background:**

The imbalance between circulating concentrations of anti- and pro-angiogenic factors is usually intense in preeclampsia with severe features (sPE). It is possible that pre-delivery circulating levels of angiogenic factors in sPE may be associated with postpartum antihypertensive drug requirements.

**Objective:**

To determine the predictive association between maternal pre-delivery serum concentrations of angiogenic factors and the use of ≥3 slow- and/or a rapid-acting antihypertensive drug therapy in sPE on postpartum days zero to three following caesarean delivery.

**Study design:**

Women with sPE (n = 50) and normotensive pregnancies (n = 90) were recruited prior to childbirth. Serum samples were obtained from each participant < 48 hours before delivery to assess the concentrations of placental growth factor (PIGF) and soluble fms-like tyrosine kinase-1 (sFlt-1) using the Roche Elecsys platform. Each participant was followed up on postpartum days zero, one, two and three to monitor BP and confirm antihypertensive treatment. The optimal cut-off thresholds of sFlt-1/PIGF ratio from receiver operating characteristic curve predictive of the antihypertensive therapy were subjected to diagnostic accuracy assessment.

**Results:**

The majority 58% (29/50) of sPE had multiple severe features of preeclampsia in the antenatal period with the commonest presentation being severe hypertension in 88% (44/50) of this group, followed by features of impending eclampsia which occurred in 42% (21/50). The median gestational age at delivery was 38 (Interquartile range, IQR 1) *vs* 36 (IQR 6) weeks, *p* < 0.001 in normotensive and sPE groups respectively. Notably, the median sFlt-1/PIGF ratio in normotensive and sPE groups were 7.3 (IQR 17.9) and 179.1 (IQR 271.2) respectively, *p* < 0.001. Of the 50 sPE participants, 34% (17/50) had early-onset preeclampsia. The median (IQR) of sFlt-1/PIGF in the early- and late-onset preeclampsia groups were 313.52 (502.25), and 166.59(195.37) respectively, *p* = 0.006. From postpartum days zero to three, 48% (24/50) of sPE received ≥ 3 slow- and/or a rapid-acting antihypertensive drug. However, the daily administration of ≥ 3 slow- and/or a rapid-acting antihypertensive drug in sPE were pre-delivery 26% (13/50), postpartum day zero 18% (9/50), postpartum day one 34% (17/50), postpartum day two 24% (12/50) and postpartum day three 20% (10/50). In sPE, the pre-delivery sFlt-1/PIGF ratio was predictive of administration of ≥3 slow- and/or a rapid-acting antihypertensive drug on postpartum days zero, one and two with the optimal cut-off ratio being ≥315.0, ≥181.5 and ≥ 267.8 respectively (sensitivity 72.7–75.0%, specificity 64.7–78.6%, positive predictive value 40.0–50.0% and negative predictive value 84.6% - 94.3%). The predictive performance of sFlt-1/PIG ratio on postpartum day 3 among the sPE was not statistically significant (area under receiver operating characteristic curve, 0.6; 95% CI, 0.3–0.8).

**Conclusion:**

A pre-delivery sFlt-1/PIGF ratio (< 181.5) is a promising predictor for excluding the need for ≥3 slow- and/or a rapid-acting antihypertensive drug therapy in the immediate postpartum period in sPE.

## Introduction

The pathogenesis of preeclampsia (PE) is not yet completely understood; however, recent evidence suggests that the disease manifests in a susceptible mother following inadequate placentation that results in abnormal placental blood flow [1, 2]. Consequentially, syncytiotrophoblast damage occurs leading to an elevation in the secretion of anti-angiogenic factors such as soluble fms-like tyrosine kinase-1 (sFlt-1) and a concurrent reduction in the levels of pro-angiogenic factors such as vascular endothelial growth (VEGF) and placental growth factors (PlGF) [3–5]. The findings of earlier studies were contributory to the current knowledge [6, 7].

The imbalance between pro- and anti-angiogenic factors in PE is known to develop long before the manifestation of the symptomatology [8] and correlates with the severity of the disease [9]. As a result, the imbalance in the circulating concentrations of angiogenic factors (sFlt-1/PIGF ratio) is usually intense in PE with severe features (i.e. severe preeclampsia, sPE) compared to those without these features [10–12]. It is established that most cases of postpartum eclampsia occur within the first 48 to 72 hours of childbirth [13, 14] and that the systemic concentration of sFlt-1 reverts to baseline within 48 to 72 hours post-delivery [3, 15]. Evidence also suggests that blood pressure (BP) is influenced by the circulating concentration of pro- and anti-angiogenic factors; PIGF reduces while sFlt-1 increases BP [16, 17]. This association between BP and angiogenic factors has been reported in women who underwent caesarean deliveries (CD) [18]. For instance, a report on singleton pregnancies delivered by CD found that antenatal circulating angiogenic factors correlate with the highest postpartum systolic and diastolic BPs [19]. Despite these findings, there are variations in the circulating concentrations of angiogenic factors amongst racial groups [20]. As a result, a clarion call to determine the circulating concentrations of angiogenic factors in different racial groups has been advocated [21] and this is of particular importance in settings with high burden of PE and diverse populations such as South Africa [22–25].

Given that the more intense the imbalance in angiogenic factors the greater the severity of the PE [11], and that antenatal circulating concentrations of these biomarkers correlate with postpartum BP, it is possible that a clinically useful predictive association exists between pre-delivery levels of angiogenic factors and postpartum BP in sPE. Undoubtedly, the administration of antihypertensive drug therapy reduces high BP. An already reduced postpartum BP may therefore not show a clinically useful predictive association with the degree of pre-delivery imbalance in angiogenic factors. Furthermore, patients with difficult-to-control hypertension usually require combined antihypertensive medications [26]. Also, the number of antihypertensive medications and their type of action (slow- or rapid-acting) depicts the severity of the hypertension and possibly the extent of imbalance in angiogenic factors. Therefore, an appropriate surrogate marker of the association between pre-delivery angiogenic imbalance and postpartum BP may be the number and type of antihypertensive medication. A finding of a predictive association between the pre-delivery angiogenic factors and postpartum antihypertensive drug requirements will assist in patient counselling and serve as a triage to ensure advance management plans (such as obstetric high care admission) for those who require ≥ 3 slow-acting and/or any rapid-acting antihypertensive medication. In South Africa, obstetric high care units are scarce resources with the number of patients that require admission therein frequently exceeding the number of available beds [27]. The majority of the patients in many of these high care units are usually those with sPE who may require ≥ 3 slow-acting and/or any rapid-acting antihypertensive medication for severe hypertension. It is possible that the pre-delivery imbalance in angiogenic factors may be valuable as a triage test to predict patients with sPE that may be managed in an ordinary hospital ward whenever the number of beds in the obstetric high care unit is insufficient.

Of note, the sFlt-1/PIGF ratio has been reported to be a better predictor of pregnancy complications than sFlt-1 or PIGF alone [28, 29], and different sFlt-1/PIGF ratios varying from ≥85 - ≥871 pg/ml have been used to predict adverse maternal outcomes in PE [30]. Unfortunately, there is no approved reference standard for the prediction of postpartum antihypertensive drug requirement. With this in mind, the aim of this study was to determine the relationship between maternal pre-delivery serum levels of angiogenic factors (sFlt/PIGF ratio) and BP on postpartum days 0–3 amongst severe preeclamptic and healthy normotensive pregnant women who had CD, using the number and type of antihypertensive medication as an outcome measure of the BP. The predelivery serum concentration of PIGF, sFlt-1 and sFlt-1/PIGF ratio in the normotensive and sPE groups were also compared.

## Materials and methods

### Study design, duration and setting

This was a prospective cohort study conducted between August—December 2015 in a regional hospital in South Africa.

### Regulatory permission

Ethical approval (reference BE236/14) to conduct the study was obtained from the Biomedical Research Ethics Committee of the University of KwaZulu-Natal, South Africa. All participants gave written informed consent prior to the study.

### Study participants

The study participants were a homogenous group of Black South Africans and included consecutive women with sPE and normotensive pregnancies who were scheduled for CD. Exclusion criteria included active phase of labour, other types of hypertensive disorders of pregnancy (such as eclampsia, PE without severe features, gestational hypertension, and chronic hypertension), diabetes, multiple pregnancy, and illness from other medical conditions. Angiogenic imbalance is usually less intense in PE without severe features [12], therefore, we excluded this group of patients.

Pre-eclampsia was defined as the development of new-onset hypertension (BP ≥140/90 mmHg) after 20 weeks of gestation with any of the following: proteinuria (≥ 300 mg in a 24 hours urine sample), fetal growth restriction and maternal organ dysfunction (renal impairment, elevated liver transaminases, haematological disorder such as thrombocytopenia, and cerebral symptoms suggestive of impending eclampsia) [31]. sPE was defined based on the presence of the following features: systolic BP ≥ 160 mmHg and or diastolic BP ≥ 110 mmHg, serum creatinine ≥ 90–110 mmol/L [31–33], elevated liver transaminases ≥ 70 IU/L [32, 34], platelet count < 100 X 10^9^/L [35], HELLP syndrome, pulmonary oedema, impending eclampsia, fetal growth restriction [36], and proteinuria ≥3 g/24 hours. The differences in the diagnostic criteria for sPE [31, 35–37] are known including the recent European guideline that recommends increased surveillance if 24 hours urine protein exceed 2g [38]. Our diagnostic criteria were supported by the high incidence [24, 25] and burden [22] of PE in our setting and its tendency to deteriorate rapidly [23]. Early onset PE (EOPE) was defined as the development of PE at < 34 weeks of gestation. Late onset PE (LOPE) was defined as the development of PE at ≥ 34 gestational weeks. The severe complications of PE as recommended by the International Society for the Study of Hypertension in Pregnancy (ISSHP) that were used to determine timing of delivery included: gestational age < 24 weeks and/or ≥34 weeks, inability to control the BP with maximum dose of three antihypertensive drugs from different classes, pulmonary oedema, HELLP syndrome, progressive deterioration in maternal condition (liver, renal, imminent eclampsia, and/or platelet count), placental abruption, and fetal compromise/demise [31].

### Data collection

Prior to delivery (< 48 hours), serum sample was collected from each participant for measurement of sFlt-1 and PIGF concentrations. The details of this process are explained in the next subheading (measurement of sFlt-1/PIGF ratio). Additionally, the BP of participants were measured during the pre-delivery period and on days 0, 1, 2 and 3 post-delivery. Day 0 refers to the day of delivery while the next consecutive three days were day 1, day 2 and day 3 post-delivery. The BP was measured using iMEC12 patient monitor (Shenzhen Mindray Bio-Medical Electronics Co., Ltd), an automated device which has passed a baseline check [39]. Each day, the BP was measured at 05:00, 08:00, 14:00 and 22:00 hours. Two measurements were taken at rest from the arm of each participant using an appropriate cuff and the BP at each point time was the average readings [40]. The antihypertensive drug therapy administered to the participants prior to delivery and on postpartum days 0–3 were also monitored and the information retrieved from the patients’ hospital charts. A dedicated and trained research midwife assisted with data collection and recording of the information in a data extraction form.

### Measurement of sFlt-1/PIGF ratio

Each participant had a peripheral venous blood sample collected using SST II advance yellow with gel tubes. The blood sample was centrifuged at 3000 rpm at room temperature for 10 minutes in a Rotina 380 R benchtop centrifuge (Andreas Hettich GmbH & Co. KG, Germany). The serum was collected and stored at -20°C. Measurement of the angiogenic factors were performed in batches within one month of specimen collection. An independent laboratory, Ampath laboratory (Durban, South Africa), determined the concentration of sFlt-1 and PIGF using the Roche Elecsys platform (Roche Diagnostics, Germany) according to the instructions of the manufacturer which were based on sandwich principle [41, 42]. The ability of sFlt-1/PIGF ratio as a predictor of the target condition (use of ≥3 slow- and/or any rapid-acting antihypertensive drug in the postpartum period) was statistically evaluated.

### Postpartum antihypertensive therapy

The treatment of the hypertension was guided by national guidelines published/endorsed by the South African department of health [40, 43]. Gradual dose reduction and final withdrawal of antihypertensive drugs were commenced in the postpartum period following normalization of the BP. The clinicians were responsible for the prescription (including the gradual withdrawal) of antihypertensives medications for the patients. Antihypertensive medication was indicated to treat postpartum BP ≥ 150/100 mmHg [32, 44]. However, BP 140-150/90-100 mmHg was treated amidst complications such as severe thrombocytopaenia (to prevent cerebrovascular accident) and renal impairment. Severe hypertension in pregnancy (BP ≥ 160/110 mmHg) is an emergency that requires rapid but controlled reduction of the high BP to achieve a target BP of 140-150/90-100 mmHg [43, 45, 46]. Therefore, the criteria that had been used to determine the use of ≥ 3 slow- and/or a rapid-acting antihypertensive drug therapy were the severity of the hypertension and the presence of other target organ complications. The antihypertensive drugs of choice for severe hypertension were intravenous labetalol or rapid-acting nifedipine. Other slow-acting antihypertensives agents were introduced sequentially to improve drug efficacy and maintain the BP control. For non-severe hypertension in the postpartum period, the commonly prescribed slow-acting antihypertensive drug was a calcium channel blocker (amlodipine or extended release nifedipine). Other slow-acting antihypertensive agents used were monohydralazine, prazosin, enalapril, alpha methyldopa, and diuretics such as hydrochlorothiazide.

### Statistical analysis

The analysis of data was performed using SPSS version 24.0 (IBM, Armonk, NY, USA) except for utilizing proc glimmix [47] in SAS 9.4 (SAS Institute Inc. Cary, NC, USA) to fit the logistic model for time-dependent receiver operating characteristic (ROC) curve. Normality in the distribution of data were assessed graphically and with the use of Shapiro-Wilk test [48]. Descriptive statistics were also performed. The differences between sPE and normotensive groups were assessed using student t-test if the data was continuous and normally distributed or using Mann-Whitney U test if the data was continuous and skewed. The differences in categorical variables between the two groups were assessed using the Chi-square test, or Fischer exact test when the frequency within a cell is <5. Repeated measures two-way analysis of variance (ANOVA) was conducted to compare the highest and lowest postpartum BPs of the two groups. The correlation between pre-delivery sFlt-1/PIGF ratio and highest postpartum BPs were assessed using Spearman’s rank correlation which is appropriate for two continuous variables with either or both being non-normally distributed [49–51]. The predictive performance of the sFlt-1/PIGF ratios were assessed using AUC (area under the curve) of ROC curves [52, 53]. Data-driven optimal cut-off threshold [49, 54] of sFlt-1/PIGF ratio was obtained at maximal Youden index (Sensitivity + Specificity - 1) [55] in the daily ROC curve coordinate. The diagnostic accuracy of the cut-off threshold was assessed and reported as positive, and negative predictive values, sensitivity, and specificity [56]. The STARD (Standards for Reporting of Diagnostic Accuracy) guideline [57] was applied in this report and the requirements in its checklist [58] satisfied.

## Results

**Fig 1:**
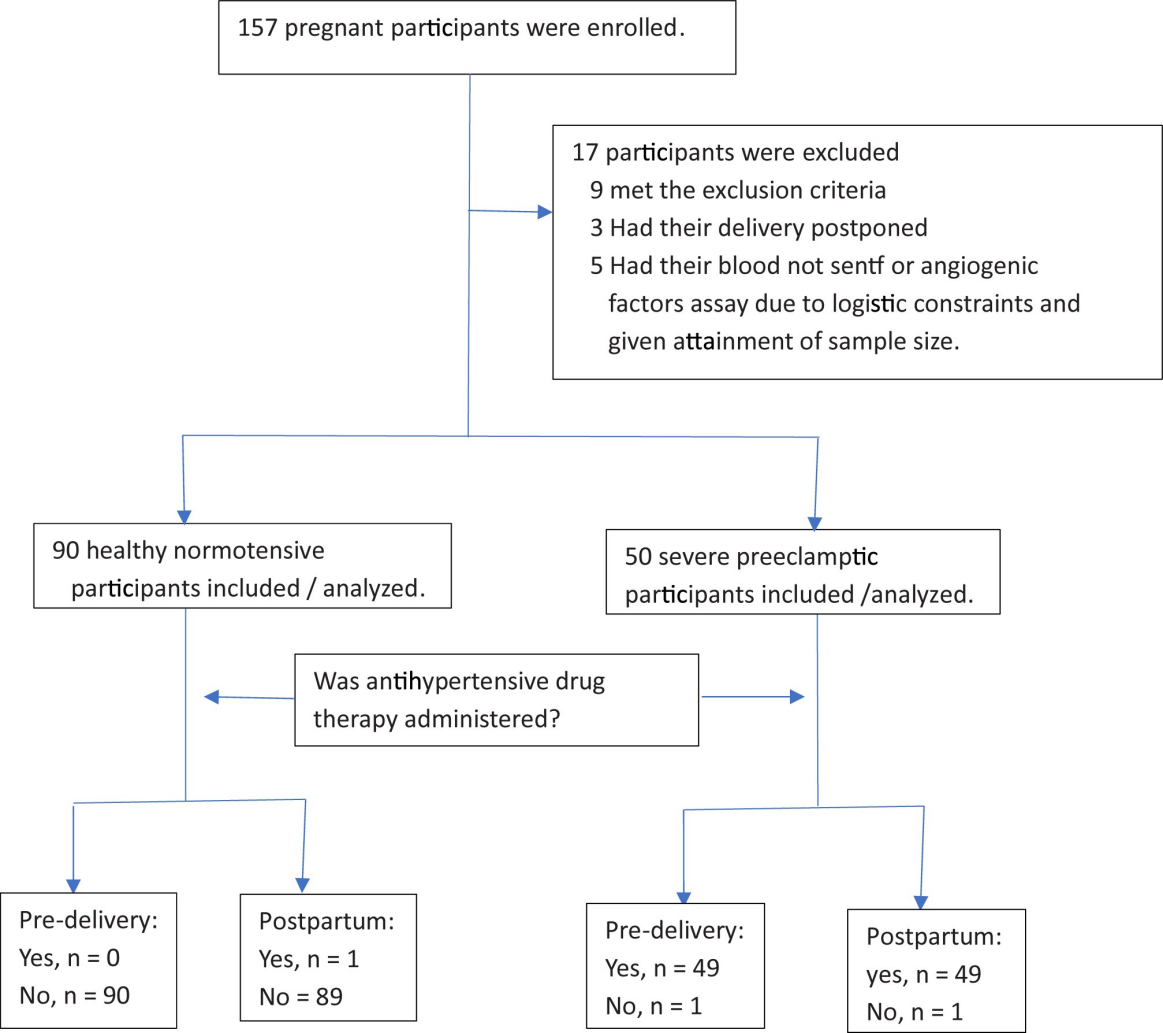
Study flow chart.

The flow diagram of the study participants is shown in Fig 1.

### Demographics

The median age of the participants was 28 (Interquartile range, IQR 7) and 23 (IQR 11) years for the normotensive and sPE groups respectively, *p* = 0.001. Primigravidae constituted 8.9% (8/90) and 46% (23/50) of the normotensive and sPE groups respectively, *p* < 0.001. The body mass index at first prenatal visit for normotensive *vs* sPE groups was 29.5 (IQR 8.3) *vs* 26.8 (IQR 6) respectively, *p* = 0.028. Of the 50 sPE participants, 17 had EOPE.

The majority (29/50) of sPE had multiple severe features with the commonest presentation being severe hypertension (44/50), followed by neurological signs and symptoms of impending eclampsia (21/50). The median gestational age at delivery was 38 (IQR 1) *vs* 36 (IQR 6) weeks, *p* < 0.001 in normotensive and sPE groups respectively. The indications for CD (fetal, maternal, and feto-maternal) in normotensive *vs* sPE were 14.4%% (13/90), 78.9% (71/90) and 6.7% (6/90) *vs* 40% (20/50), 46% (23/50), and 14% (7/50) respectively, *p* < 0.001.

### Prenatal supplementation

Prenatal aspirin therapy to prevent PE was received by 2.2% (2/90) and 4% (2/50) of normotensive and sPE respectively, *p* = 0.67. Additionally, prenatal calcium supplementation for prevention of PE was administered to 94.4% (85/90) and 84% (42/50) of the normotensive and sPE groups respectively, *p* = 0.041.

### sFlt-1/PIGF ratio

The population reference values of angiogenic factors at different weeks of gestation before delivery (guided by the time of peak and trough of circulating concentrations of sFlt-1 and PIGF [21], and also by the categorization of PE into early- and late-onset disease, as well as classification of pregnancies into term and post-term) is shown in Table 1. There were no missing data. The median sFlt-1/PIGF ratio in normotensive and sPE groups were 7.3 (IQR 17.9) and 179.1 (IQR 271.2) respectively, *p* < 0.001. Further assessment showed that the sFlt-1/PIGF ratio of the two normotensive participants on aspirin were 38.55 and 6.95. On the other hand, the sFlt-1/PIGF ratio of the two sPE participants on aspirin were 117.79 and 90.44.

**Table 1:** Serum concentration of angiogenic factors at different weeks of gestation.

Gestational age in weeks [and number of participants]	Levels	Angiogenic factor concentration (pg/ml) at various gestational age intervals					
		Normotensive, n = 90			Severe pre-eclampsia, n = 50		
		sFlt-1	PIGF	sFlt-1/PIGF ratio	sFlt-1	PIGF	sFlt-1/PIGF ratio
23 weeks [normotensive = 0; sPE = 1]	Minimum	NP	NP	NP	NA	NA	NA
	Median (IQR)	NP	NP	NP	15533.00 (NA)	23.03 (NA)	675.34 (NA)
	Maximum	NP	NP	NP	NA	NA	NA
24–28 weeks [normotensive = 0; sPE = 5]	Minimum	NP	NP	NP	8541.00	16.09	227.08
	Median (IQR)	NP	NP	NP	12341.00 (39722.5)	26.28 (32.19)	628.36 (889.96)
	Maximum	NP	NP	NP	81940.00	59.34	1380.86
29–33 weeks [normotensive = 0; sPE = 11]	Minimum	NP	NP	NP	7771.00	17.18	50.66
	Median (IQR)	NP	NP	NP	12290.00 (5222.00)	46.68 (95.71)	268.54 (411.88)
	Maximum	NP	NP	NP	21275.00	209.20	1238.36
34–36 weeks [normotensive = 6; sPE = 11]	Minimum	1686.00	214.90	1.01	5962.00	36.66	58.46
	Median (IQR)	2663.50 (1968.80)	230.75 (960.88)	11.67 (14.32)	12611.00 (10260.00)	59.03 (58.73)	183.37 (221.08)
	Maximum	3759.00	1675.50	17.24	21544.00	120.00	474.99
37–40 weeks [normotensive = 71; sPE = 19]	Minimum	1116.00	68.00	1.66	1264.00	32.89	10.28
	Median (IQR)	3482.00 (2622.00)	453.50 (711.60)	5.98 (17.45)	10768.00 (7038.00)	98.78 (93.32)	115.16 (187.49)
	Maximum	14071.00	2427.00	166.84	27807.00	204.60	525.72
41–42 weeks [normotensive = 13; sPE = 3]	Minimum	2596.00	102.35	1.67	6350.00	49.29	48.92
	Median (IQR)	5174.00 (2532.00)	211.80 (536.75)	20.61 (29.36)	10849.00 (NA)	64.93 (NA)	167.09 (NA)
	Maximum	8443.50	1622.00	82.50	21098	129.80	428.04

In each gestational age category ≥34 weeks, the comparison between normotensive and sPE groups for each of sFlt-1, PIGF and sFlt-1/PIGF ratio was statistically significant with *p*-value <0.026. Abbreviations: NA, not applicable; NP, no participant; IQR, interquartile range; PIGF, placental growth factor; sPE, preeclampsia with severe features; sFlt-1, soluble fms-like tyrosine kinase-1.

### Postpartum blood pressure and sFlt-1/PIGF ratio

The highest and lowest BPs on postpartum days 0–3 are illustrated in Table 2. Additionally, Table 3 shows the correlation between predelivery sFlt-1/PIGF ratio and the mean of highest and lowest blood pressures on days 0–3 postpartum. When the ability of sFlt-1/PIGF ratio to predict severe systolic and diastolic hypertension on days 0–3 postpartum were assessed, the AUC was 0.77–0.88, *p* < 0.05 in both groups and 0.36–0.7, *p* > 0.05 in sPE.

**Table 2:** Highest and lowest daily postpartum blood pressures.

Postpartum day	Mean of highest postpartum BP (mmHg)				Mean lowest postpartum BP (mmHg)			
	Normotensive		sPE		Normotensive		sPE	
	^a^Systolic BP	^b^Diastolic BP	^a^Systolic BP	^b^Diastolic BP	^c^Systolic BP	^d^Diastolic BP	^c^Systolic BP	^d^Diastolic BP
Day 0	125.62 ± 14.58	74.91 ± 9.64	149.13 ± 18.26	91.24 ± 12.77	104.28 ± 11.73	56.31 ± 9.34	119.30 ± 18.42	68.22 ± 13.25
Day 1	122.53 ± 13.24	75.21 ± 9.20	148.06 ± 15.73	94.68 ± 10.53	102.93 ± 11.16	59.10 ± 9.62	114.84 ± 14.59	68.08 ± 11.32
Day 2	119.32 ± 14.48	74.75 ± 10.70	151.14 ± 13.82	93.98 ± 19.03	103.80 ± 11.31	60.64 ± 9.39	126.29 ± 15.30	74.76 ± 12.26
Day 3	118.71 ± 12.25	76.64 ± 10.70	157.92 ± 14.10	101.41 ± 12.18	109.45 ± 10.81	63.95 ± 9.08	128.39 ± 17.97	80.68 ± 13.93

Abbreviations: BP, Blood pressure; sPE, Preeclampsia with severe features.

^a^Highest systolic BP: Time had no significant effect on highest systolic BP (F-ratio 0.73, *p* = 0.50, partial ecta squared [pη^2^] = 0.01) while participant group had a significant effect, (F-ratio 130.96, *p* < 0.00.1, pη^2^ = 0.65).

^b^Highest diastolic BP: There was significant effect of time (F-ratio 6.95, *p* < 0.001, pη^2^ = 0.09) and participant group (F-ratio 125.91, *p* < 0.001, pη^2^ = 0.64) on highest diastolic BP. The significant difference with time was noted between postpartum days 0 and 3, days 1 and 3, and days 2 and 3.

^c^Lowest systolic BP: Time (F-ratio 11.49, *p* < 0.001, pη^2^ = 0.14) and participant group (F-ratio 60.05, *p* < 0.001, pη^2^ = 0.46) had significant effect on lowest systolic BP. The significant difference with time was observed between postpartum days 0 and 3, days 1 and 2, days 1 and 3, and days 2 and 3.

^d^Lowest diastolic BP: There was significant effect of time (F-ratio 12.82, *p* < 0.001, pη^2^ = 0.15) and participant group (F-ratio 49.21, *p* < 0.001, pη^2^ = 0.41) on lowest diastolic BP. The significant difference with time was noted between postpartum days 0 and 3, days 1 and 3, and days 2 and 3.

**Table 3:** Correlation between predelivery sFlt-1/PIGF ratio and the mean of highest or lowest blood pressures on days 0–3 postpartum.

Mean postpartum BP	Spearman’s correlation					
	Normotensive and sPE groups, n = 140		Normotensive, n = 90		sPE, n = 50	
	Correlation coefficient	*p*-value	Correlation coefficient	*p*-value	Correlation coefficient	*p*-value
Highest systolic BP	+ 0.658	<0.001^a^	+ 0.136	0.200	+ 0.077	0.596
Highest diastolic BP	+ 0.647	<0.001^a^	+ 0.168	0.114	+ 0.217	0.131
Lowest systolic BP	+ 0.559	<0.001^a^	+ 0.276	0.008^a^	+ 0.101	0.487
Lowest diastolic BP	+ 0.548	<0.001^a^	+ 0.143	0.179	+ 0.046	0.749

Abbreviation: sPE, Preeclampsia with severe features.

^a^Significant *p*-value.

### Postpartum antihypertensive drug therapy and sFlt-1/PIGF ratio

Of the sPE, 48% (24/50) received ≥ 3 slow- and/or rapid-acting antihypertensive drug therapy on days 0–3 postpartum. One normotensive participant in the pre-delivery period developed sustained hypertension postpartum and received < 3 slow-acting antihypertensive drugs and no rapid-acting antihypertensive agent. Further details on antihypertensive drug therapy are provided in Table 4. No normotensive participant received antihypertensive therapy in the pre-delivery period. The median (IQR) of sFlt-1/PIGF ratio in participants who received (n = 24) *vs* those who did not receive (n = 116) ≥ 3 slow- and/or a rapid-acting antihypertensive drug on any of postpartum days 0–3 were 267.83 (299.96) *vs* 13.97 (45.31), *p* < 0.001 respectively in both groups (sPE and normotensive). The sFlt-1/PIGF ratio of the “normotensive” patient who received < 3 slow-acting antihypertensive drugs in the postpartum period was 5.61.

**Table 4:** Antihypertensive drug therapy in preeclampsia with severe features.

Category of antihypertensive drug	Number (%) of preeclampsia with severe features, n = 50
Pre-delivery	
None	1(2)
1–2 non-rapid acting oral agent	36(72)
≥3 oral agent or ≥1 rapid acting agent	13(26)
Postpartum day 0	
None	2(4)
1–2 non-rapid acting oral agent	39(78)
≥3 oral agent or ≥1 rapid acting agent	9(18)
Postpartum day 1	
None	9(18)
1–2 non-rapid acting oral agent	24(48)
≥3 oral agent or ≥1 rapid acting agent	17(34)
Postpartum day 2	
None	11(22)
1–2 non-rapid acting oral agent	27(54)
≥3 oral agent or ≥1 rapid acting agent	12(24)
Postpartum day 3	
None	18(36)
1–2 non-rapid acting oral agent	18(36)
≥3 oral agent or ≥1 rapid acting agent	10(20)
Missing data	4(8)

**Fig 2:**
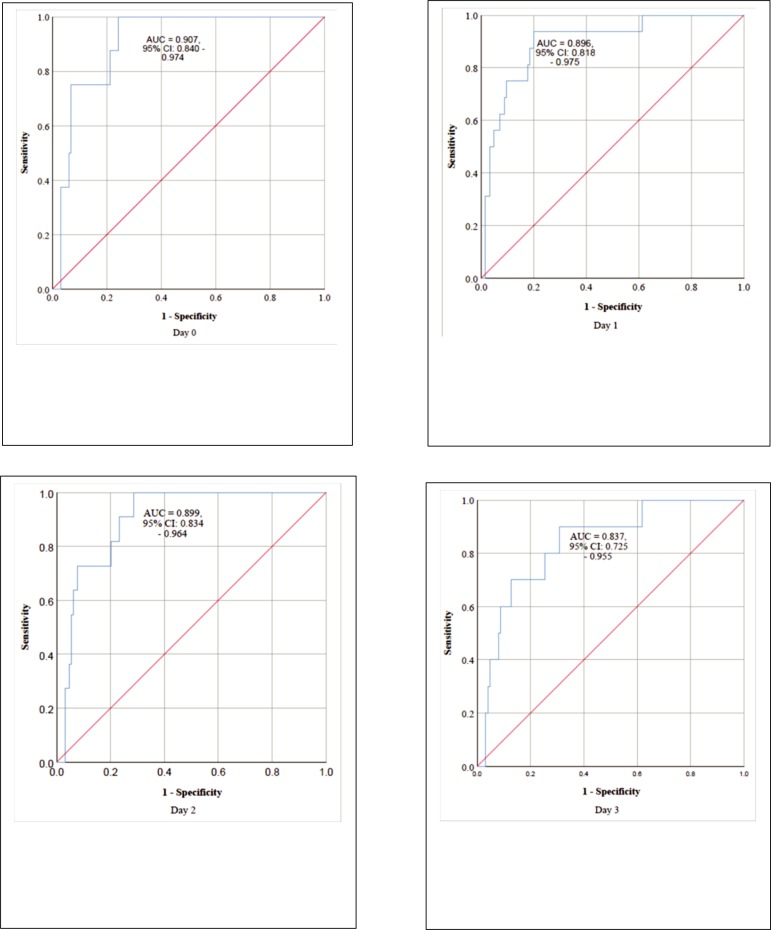
Area under receiver operating characteristic curve (AUC) showing the performance of sFlt-1/PIGF ratio in predicting the administration of ≥ 3 slow- and/or a rapid-acting antihypertensive drug on postpartum Day 0, Day 1, Day 2 and Day 3 in both groups of women with preeclampsia with severe features and normotensive pregnancy.

**Fig 3:**
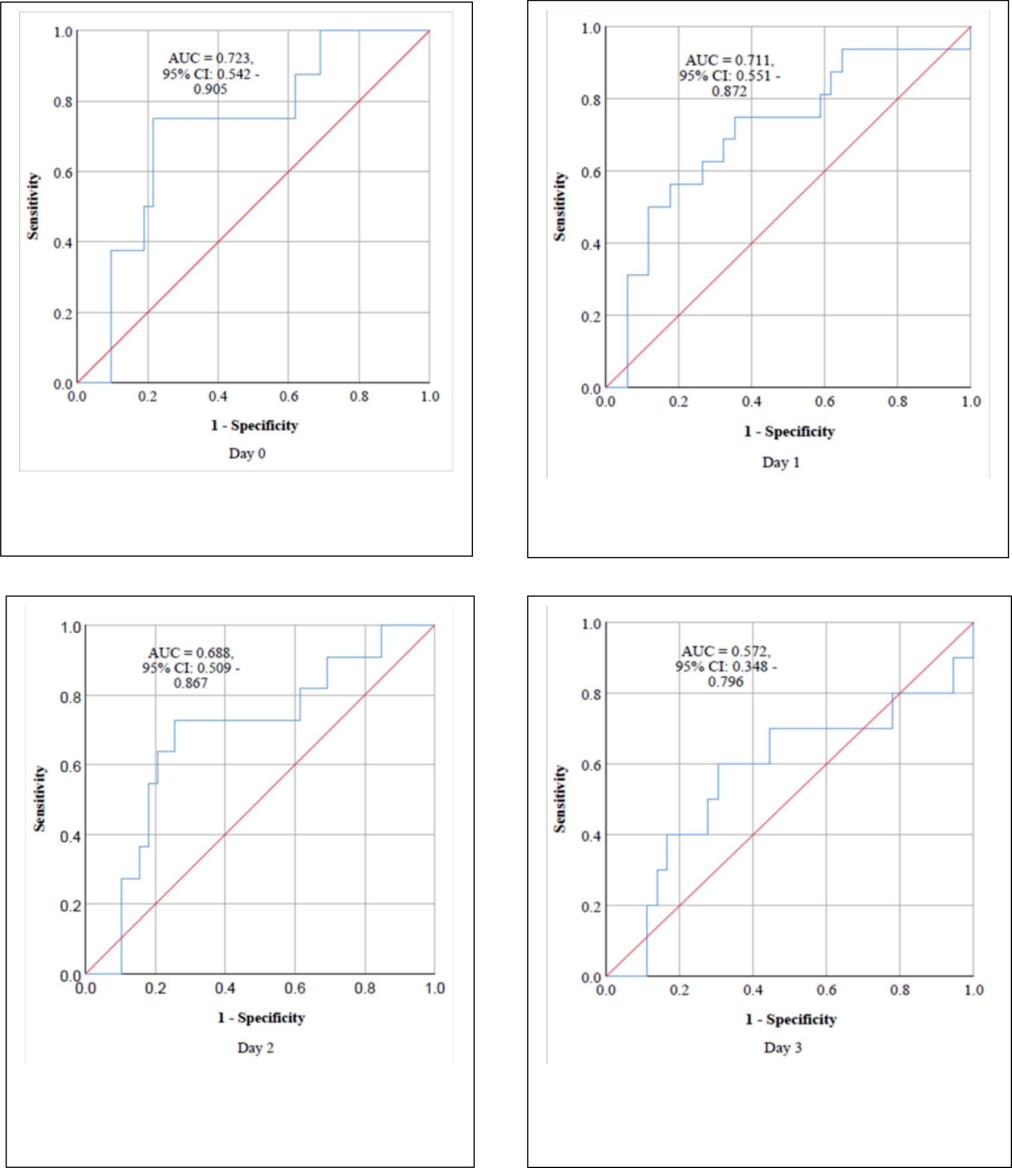
Area under receiver operating characteristic curve (AUC) showing the performance of sFlt-1/PIGF ratio in predicting the administration of ≥ 3 slow- and/or a rapid-acting antihypertensive drug on postpartum Day 0, Day 1, Day 2 and Day 3 in women with preeclampsia with severe features.

The ROC curve in Fig 2 shows the ability of sFlt-1/PIGF ratio to predict participants who had ≥3 slow- and/or a rapid-acting antihypertensive drug on postpartum days 0, 1, 2 and 3 in both the normotensive and sPE groups combined together, n = 140. In sPE group, the performance of sFlt-1/PIGF ratio in predicting the use of ≥3 slow- and/or a rapid-acting antihypertensive agent on days 0–3 postpartum is shown in Fig 3 with the area under ROC curve on day 3 being 0.6 (95% CI, 0.3–0.8) indicating a non-statistically significant AUC because the confidence interval includes 0.5 (null hypothesis = AUC of 0.5). Again, there was no participant in the normotensive group that received ≥3 slow- and/or rapid-acting antihypertensive agents. Therefore, the predictive ability of sFlt-1/PIGF ratio in the normotensive group alone could not be assessed. The area under the time-dependent ROC curves showed: both groups 0.617, *p* <0.001; sPE 0.519, *p* = 0.088; but the normotensive group did not fit the model. The diagnostic accuracy of the optimal cut-off values of sFlt-1/PIGF ratios are shown in Table 5. For purposes of completeness, the diagnostic accuracy of the sFl-1/PIF ratio among sPE on day 3 was calculated despite the confidence interval of the AUC on day 3 that included 0.5 (Fig 3). Nonetheless, when the sPE group was sub-categorized, the sFlt-1/PIGF ratio did not demonstrate optimal ability to predict postpartum antihypertensive requirements in EOPE (AUC 0.50–0.61, *p* > 0.05) and in LOPE (AUC 0.57–0.78, *p* > 0.05). Importantly, the median (IQR) of sFlt-1/PIGF in the EOPE and LOPE groups were 313.52 (502.25), and 166.59(195.37) respectively, *p* = 0.006.

**Table 5:** Performance of sFlt-1/PIGF ratio in predicting administration of ≥3 slow- and/or rapid-acting antihypertensive agents in the postpartum period.

Patient category and postpartum day	Optimal cut-off value of sFlt-1/PIGF	Sensitivity (%)	Specificity (%)	Positive predictive value (%)	Negative predictive value (%)	Risk ratio	
						RR	*P*-value (CI)
Both groups							
Day 0	≥86.5	100	75.8	20.0	100	41.9	0.010 (2.5–709.0)
Day 1	≥86.5	93.8	79.8	37.5	99.0	37.5	<0.001 (5.1–274.5)
Day 2	≥81.3	90.9	76.0	24.4	99.0	24.1	0.002 (3.2–182.6)
Day 3	≥61.7	80.0	73.8	19.5	97.9	9.3	0.004 (2.1–41.8)
sPE group							
Day 0	≥315.0	75.0	78.6	40.0	94.3	7.0	0.010 (1.6–30.8)
Day 1	≥181.5	75.0	64.7	50.0	84.6	3.3	0.019 (1.2–8.7)
Day 2	≥267.8	72.7	74.4	44.4	90.6	4.7	0.011 (1.4–15.7)
Day 3	≥257.6	60.0	69.4	35.3	86.2	2.6	0.099 (0.8–7.8)

Abbreviations: Both groups, Normotensive and sPE groups; CI, Confidence interval; RR, Relative risk; sPE, preeclampsia with severe features.

## Discussion

Angiogenic imbalance (sFlt-1/PIGF ratio) was high in sPE compared to the normotensive group with the median of the ratios being 179.1 *vs* 7.3 respectively. The elevated sFlt-1 and low PIGF levels in the sPE group is similar to findings in other studies. For instance, a previous study from a high income country (the United States) [12] reported similar results. Another study in an upper middle income country (Mexico), similar to South Africa in income ranking, also showed an elevated sFlt-1/PIGF ratio in sPE [11]. Novel therapies used in managing PE aim at reversing this angiogenic imbalance [21, 59, 60] which is usually worse in EOPE than LOPE [61] as demonstrated in the present study. Importantly, significant angiogenic imbalance occurs in both EOPE and LOPE and this explains the use of sFlt-1/PIGF ratio ≥ 85 and ≥110 to diagnose EOPE and LOPE respectively [21, 62–64] when there is clinical suspicion but doubtful diagnosis (with the greatest ability of the test being its high negative predictive value) [65, 66]. Of the 50 sPE participants, 34% (17/50) had EOPE. This is similar to findings of other studies: EOPE 27.6% *vs* LOPE 72.4% [67], and EOPE 35.5% *vs* LOPE 64.5% [68]. In susceptible women, EOPE occurs due to inadequate remodelling of spiral arteries while LOPE develops because the placenta overgrows its blood supply or becomes senescent. In each case, syncytiotrophoblastic stress occurs and leads to increased secretion of pro-inflammatory mediators such as sFlt-1 that propagate the clinical features of the disease [69, 70].

In the present study, severe hypertension was the commonest presenting clinical feature in 88% (44/50) of sPE. In the same group (sPE), 46% (23/50) had CD due to maternal indications. In a previous study, the frequency of maternal indications for delivery, mainly severe hypertension, exceeded fetal indications [71]. The predominance of maternal indications for delivery may be a reflection of the maternal severity of the PE which has been reported to correlate with the level of sFlt-1/PIGF ratio [9].

The present study demonstrates that in sPE, pre-delivery sFlt-1/PIGF ratio of ≥ 315.0, ≥ 181.5, ≥ 267.8 and ≥ 257.6 have high negative predictive value for administration of ≥ 3 slow- and/or a rapid-acting antihypertensive agents on postpartum days 0–3 respectively following CD; although this was not statistically significant on postpartum day 3 (as the confidence interval of the area under the ROC curve on day 3 [Fig 3] includes the null point of 0.5) [49] possibly due to the return of sFlt-1 to pre-pregnancy levels within 48–72 hours postpartum. Additionally, in the combined group of sPE and normotensive pregnant women who had CD, pre-delivery sFlt-1/PIGF ratio of ≥ 86.5, ≥ 86.5, ≥ 81.3 and ≥ 61.7 have high negative predictive value to predict administration of ≥ 3 slow- and/or a rapid-acting antihypertensive drug therapy on postpartum days 0–3 respectively. The intended use of this test is for prediction of postpartum antihypertensive requirement but our findings show that the negative predictive value is better than the positive predictive value. The clinical role of the test is to triage pregnant women and make provision for the antihypertensive needs of those at increased risk of requiring ≥3 slow- and/or rapid-acting antihypertensive drug therapy. Although there is a tendency towards normalization of BP after delivery of the baby and placenta in PE, some women in the puerperium may develop complications of hypertension if adequate plans are not made for their postpartum antihypertensive needs. Therefore, this screening test has a great potential in clinical practice as cost savings may accrue because triage will be possible, and low-risk women (at decreased risk of requiring ≥ 3 slow- and/or a rapid-acting antihypertensive agent) will require non-intensive monitoring and incur less cost.

The sFlt-1/PIGF ratios predictive of the postpartum antihypertensive requirement in the present study are within the range previously found to be associated with adverse maternal outcomes [30]. Previously, PIGF (≤0.4 - ≤122 pg/ml) and sFlt-1/PIGF ratios (≥85 - ≥871) have been applied to predict adverse maternal outcomes in PE [30]. Nonetheless, an sFlt-1/PIGF ratio above 655 was found to be non-predictive of impaired perinatal outcome, but the authors suggest that levels above 1000 may be useful [72]. Although PIGF may not be as good as sFlt-1/PIGF ratio, a previous study has shown that with or without PE, low PIGF (below 100 pg/ml) levels signal increased risk of adverse outcomes of pregnancy [73]. Unsurprisingly in the present study, there was a sub-optimal performance [53, 54] by sFlt-1/PIGF ratio in predicting severe postpartum systolic and diastolic hypertension in sPE probably because antihypertensive medications may have modified the BP levels. The complex heterogenous pathogenesis of PE [21, 74, 75] may also account for this finding.

Nonetheless, to the best of our knowledge, there is no previous study on prediction of postpartum antihypertensive drug requirements. Understandably, the prediction of pregnancy events in PE using angiogenic factors is influenced by the complex pathogenesis of the disease as well as the absent or less pronounced angiogenic imbalance in some cases called “non-angiogenic” PE (which is prevalent in LOPE associated with comorbidity such as obesity) [76]. It is possible, however, that indices such as sFlt-1/PIGF ratio, serum NT-proBNP (N-Terminal Prohormone of Brain Natriuretic Peptide), and total peripheral resistance which are predictive of hypertensive disorders of pregnancy [77] may predict antihypertensive drug requirement. Possibly, a combination of multiple factors that includes the pre-delivery sFlt-1/PIGF ratio may improve the prediction of postpartum antihypertensive medications and may even act as a marker of resistant hypertension. These require further investigation.

### Strengths and limitations

The categorization of the duration of data collection into postpartum day 0, day 1, day 2 and day 3 may influence the number of BP measurements performed on postpartum day 0 and consequentially affect the day 0 mean BP. This is because the CD were performed at different time points as determined by the clinical indications. It will be unethical and harmful to defer an emergency CD of a viable fetus for the purposes of this study. Therefore, our approach resembles the practical situation in most clinical settings where a day of hospital stay is from 00:00 to 23:59 hours. Furthermore, apart from sFlt-1 and PIGF, factors such as fluid therapy, administration of vasoactive medications and psychological stress, to mention but a few, affect postpartum BP [17, 69, 78, 79]. Although complex, it will be beneficial for future studies to investigate the contributions of these factors in determining postpartum BP levels. Such studies may also assist to determine if there is a time lag between postpartum resolution of angiogenic imbalance and the return of BP to pre-pregnancy level.

Given the lack of an inter-class dose (efficacy) equivalent table of different types of antihypertensive agents, we did not include the strength/dosage of antihypertensive agents administered to the research participants. Regardless, there is variability in BP control response to an antihypertensive agent, with extra medication from another class of antihypertensive agent added to the drug regimen of an individual with poorly controlled hypertension. In the antepartum period, however, one is apprehensive of the possible effects of combined antihypertensive medications on the foetus but it is prudent to control BP in pregnancy and postpartum period to avert adverse outcomes such as stroke. Despite the scarcity of robust data to direct the drug treatment of hypertension during pregnancy and the puerperium [80], women in the index study were only treated with the commonly recommended antihypertensive agents with long history of safety in pregnancy.

Due to lack of hospital bed-space [81], and local challenges associated with follow-up of outpatients for research purposes it was not feasible to include women who had vaginal deliveries as research participants. The challenges with follow-up of patients also affect other disciplines in South Africa. For instance, a recent study in South Africa indicates that after a surgery for ankle fracture, 6/268 (3.3%) of the patients attended all the follow-up clinic visits while 56/268 (20.9%) did not attend any [82]. We speculate that unavailability of transport to the clinic, change of personal telephone numbers, unaffordability or unsteady use of a specified family physician, and change of residential address which makes home visit challenging are additional realities in our setting. Our feasibility study prior to the study, therefore, did not support long-term patient follow-up for the purposes of this research. Importantly, a study in the United States also indicates that the postpartum follow-up rate after sPE was 52% [83]. In our opinion, the follow-up rate for HDP is challenging because hypertension is a silent killer–may not cause any symptoms initially but results in a target organ damage later.

Additionally, the administration of prophylactic calcium and or aspirin may have caused treatment paradox [84] that modified the effect of the risk factors on the development and outcomes of PE among the participants. The possible alteration of the circulating levels of angiogenic factors by aspirin reported in a previous study [85] is noted. However, only two normotensive and two sPE patients were on aspirin. The sFlt-1/PIGF ratios of these four participants were not consistently lower or higher than the median value of their respective groups (normotensive or sPE as applicable). Understandably, it may be argued that the aspirin therapy could have altered the levels of the sFlt-1/PIGF ratios. Most importantly, a recent study where aspirin was efficacious in preventing adverse pregnancy outcomes found the levels of angiogenic factors to be similar in users and non-users of aspirin [86]. The interpretation of this finding according to the investigators was that aspirin could be efficacious through other pathways other than altering the levels of angiogenic factors [86]. Another recent study did not find an association between every form of adherence to aspirin therapy and abnormal angiogenic markers [87]. Therefore, future studies are required to further investigate the effect of aspirin on circulating levels of angiogenic factors in different racial/population groups. Whether or not prenatal calcium supplementation alters the levels of angiogenic factors in pregnancy is largely unknown and requires further investigation in future studies. Nonetheless, the authors of the present study also note that there is a paucity of studies that indicate whether and to what extent antihypertensive medications affect the circulating level of angiogenic factors in PE. Notably, antihypertensive medications do not alter the placental biosynthesis and or secretion of angiogenic factors in PE [88], but undoubtedly reduce BP levels.

Notably, due to poor distribution of numbers within groups, we were unable to calculate the predictive cut-off thresholds of sFlt-1/PIGF ratio and their diagnostic accuracies in sub-groups of the participants. To explain, there was no participant in the normotensive group that received ≥ 3 slow- and/or rapid-acting antihypertensive agents, and as a result, the predictive ability of sFlt-1/PIGF ratio in the normotensive group alone could not be assessed. Therefore, future investigators of this topic should increase the sample size utilizing the findings of the present study in the power calculation to ensure that sufficient number of participants with EOPE and those with LOPE are sampled to optimize both the sensitivity and specificity. Unfortunately, the time duration of such a study and the cost implications may be an impediment, particularly in resource-constrained countries like South Africa. Additionally, the generalizability of the present study (even to patients having vaginal deliveries) is still limited until follow-up validation studies are conducted. To this end, plans are underway to commence the validation study.

The strength of this study includes the establishment of a much-needed optimal threshold of sFlt-1/PIGF ratio for predicting antihypertensive drug usage in the immediate postpartum period. Notably, hypertension is only second to Human Immunodeficiency Virus related illnesses as a cause of mortality in adults in Africa [89]. Therefore, the study addresses a research priority in the African continent being an observational study focused on events in the postpartum period after operative delivery [90]. To the best of our knowledge, the present study is the first of its kind to provide the cut-off thresholds of pre-delivery sFlt-1/PIGF ratio that may be utilized to predict the use of antihypertensive medications in the postpartum period in normotensive pregnancy and sPE.

## Conclusion

The clinical management of sPE may be improved by utilizing sFlt-1/PIGF ratio to predict the antihypertensive requirements in the immediate postpartum period. Future large-scale studies are required to validate this finding.

## Supporting information

S1 Table The 2015 STARD (Standards for Reporting of Diagnostic Accuracy) 30-item checklist. (DOCX)

S2 Table Statistically significant difference in the pre-delivery serum concentration of angiogenic factors between normotensive pregnancy and preeclampsia with severe features. (DOCX)
