# Supplementary material for: The performance of pre-delivery serum concentrations of angiogenic factors in predicting postpartum antihypertensive drug therapy following abdominal delivery in severe preeclampsia and normotensive pregnancy
Source: PLoS One. 2019 Apr 25;14(4):e0215807. doi: 10.1371/journal.pone.0215807 (PMC6485032; doi:10.1371/journal.pone.0215807)
Supplement: S1 Table — (DOCX) [file pone.0215807.s001.docx]

S1 Table. The 2015 STARD (Standards for Reporting of Diagnostic Accuracy) 30-item checklist. Available at: <https://www.equator-network.org/reporting-guidelines/stard/>

| **Section** | **STARD Item No** | **Recommended STARD item** | **Manuscript section where recommended item was addressed** |
| --- | --- | --- | --- |
| TITLE OR ABSTRACT |  |  |  |
|  | 1 | Identification as a study of diagnostic accuracy using at least one measure of accuracy (such as sensitivity, specificity, predictive values, or AUC) | Title: It contains the word ‘predict’ to identify the report as a diagnostic study.  Abstract: Results were reported to include sensitivity, specificity and predictive values. |
| ABSTRACT |  |  |  |
|  | 2 | Structured summary of study design, methods, results, and conclusions (for specific guidance, see STARD for Abstracts) | The abstract is structured. |
| INTRODUCTION |  |  |  |
|  | 3 | Scientific and clinical background, including the intended use and clinical role of the index test | Introduction: First, second and third paragraphs contain the scientific and clinical background. The third paragraph also describes the intended use as prediction and clinical use as triage. |
|  | 4 | Study objectives and hypotheses | Introduction: The last sentence of the introduction section contains the objective of the study. |
| METHODS |  |  |  |
| Study design | 5 | Whether data collection was planned before the index test and reference standard were performed (prospective study) or after (retrospective study) | Materials and Methods: In the first paragraph, the study is described as prospective. |
| Participant | 6 | Eligibility criteria | Materials and Methods, sub-section on study participant contains the eligibility criteria. |
|  | 7 | On what basis potentially eligible participants were identified (such as symptoms, results from previous tests, inclusion in registry) | Materials and Method, sub-section on study participant: The participants were identified as severe preeclamptic and normotensive pregnant women scheduled for caesarean delivery. This subsection contains the definition of preeclampsia with severe features. |
|  | 8 | Where and when potentially eligible participants were identified (setting, location and dates) | Materials and Methods, Study design, duration and setting:  Contains the setting, location and date. |
|  | 9 | Whether participants formed a consecutive, random or convenience series | Materials and Methods, study participant: Indicates that the participants were consecutive series. |
| Test methods | 10a | Index test, in sufficient detail to allow replication | Materials and Methods, measurement of sFlt-1/PIGF ratio: Contains the index test. |
|  | 10b | Reference standard, in sufficient detail to allow replication | There is no reference standard as indicated in the last paragraph of the introduction section. |
|  | 11 | Rationale for choosing the reference standard (if alternatives exist) | There is no reference standard as indicated in the last paragraph of the introduction section. |
|  | 12a | Definition of and rationale for test positivity cut-offs or result categories of the index test, distinguishing pre-specified from exploratory | The introduction section contains the rational for the test  Materials and Methods: The study is novel. |
|  | 12b | Definition of and rationale for test positivity cut-offs or result categories of the reference standard, distinguishing pre-specified from exploratory | There is no reference standard as indicated in the last paragraph of the introduction section. |
|  | 13a | Whether clinical information and reference standard results were available to the performers/readers of the index test | The laboratory staff was blinded from participants diagnoses and any specimen source information. |
|  | 13b | Whether clinical information and index test results were available to the assessors of the reference standard | There is no reference standard as indicated in the last paragraph of the introduction section. |
| Analysis | 14 | Methods for estimating or comparing measures of diagnostic accuracy | Statistical Analysis: Receiver operating characteristic curves were used to determine the discriminating thresholds. It also contains the types of diagnostic accuracy assessments that were performed. |
|  | 15 | How indeterminate index test or reference standard results were handled | There was no indeterminate index test. The index test is a numerical data (sFlt-1/PIGF ratio) measured in a laboratory. |
|  | 16 | How missing data on the index test and reference standard were handled | Discussion: There was no missing data as indicated in the sFlt-1/PIGF ratio sub-section. |
|  | 17 | Any analyses of variability in diagnostic accuracy, distinguishing pre-specified from exploratory | Variability in the diagnostic accuracy were calculated. For instance, confidence intervals are included in Table 3. |
|  | 18 | Intended sample size and how it was determined | The sample population were 50 women with preeclampsia with severe features and 90 normotensive pregnant women. The sample size for the present study was estimated using a table^a^ that contains the output of Power Analysis and Sample Size (PASS) software (PASS 11. NCSS, LLC. Kaysville, Utah, USA). The NCSS PASS software is reliable and widely accepted for use to determine the sample size of sensitivity and specificity studies.^b^  Reference for sample size estimation:   1. Bujang MA, Adnan TH. Requirements for Minimum Sample Size for Sensitivity and Specificity Analysis. J Clin Diagn Res 2016;10(10):YE01-YE6. <http://dx.doi.org/10.7860/JCDR/2016/18129.8744>. 2. Malhotra RK. Sample Size Considerations for Diagnostic Tests: Application to Sensitivity and Specificity. In: Doi SAR, Williams GM, editors. Methods of Clinical Epidemiology. Heidelberg, Germany: Springer; 2013. <http://dx.doi.org/10.1007/978-3-642-37131-8>. |
| RESULTS |  |  |  |
| Participants | 19 | Flow of participants, using a diagram | Results: This is shown in Figure 1. |
|  | 20 | Baseline demographic and clinical characteristics of participants | Results: This is contained in the demographics sub-section. |
|  | 21a | Distribution of severity of disease in those with the target condition | Results: This is contained in the second paragraph of demographics. |
|  | 21b | Distribution of alternative diagnoses in those without the target condition | Patients with other diagnoses were excluded. |
|  | 22 | Time interval and any clinical interventions between index test and reference standard | Blood sample for index test was collected 24 – 48 hours before delivery, as indicated in the sub-section data collection under Materials and Methods.  There is no reference standard. |
| Test results | 23 | Cross tabulation of the index test results (or their distribution) by the results of the reference standard | Results: Distribution of the index test is shown in Table 1 and in the following sub-sections of the results:   1. sFlt-1/PIGF ratio. 2. Postpartum blood pressure and sFlt-1/PIGF ratio. 3. Postpartum antihypertensive drug therapy and sFlt-1/PIGF ratio. |
|  | 24 | Estimates of diagnostic accuracy and their precision (such as 95% confidence intervals) | Figure 1, Figure 2, and Table 3 |
|  | 25 | Any adverse events from performing the index test or the reference standard | Peripheral vein was as the source of blood sample collection and there was no adverse event. |
| DISCUSSION |  |  |  |
|  | 26 | Study limitations, including sources of potential bias, statistical uncertainty, and generalisability | Strengths and limitations are discussed under this sub-heading. |
|  | 27 | Implications for practice, including the intended use and clinical role of the index test | Discussion: third paragraph |
| OTHER INFORMATION |  |  |  |
|  | 28 | Registration number and name of registry | Reference BE236/14 was assigned to the study protocol by the Biomedical Research Ethics Committee of the University of KwaZulu-Natal, South Africa.  The study is not registered else where. |
|  | 29 | Where the full study protocol can be accessed | University of KwaZulu-Natal, South Africa. |
|  | 30 | Sources of funding and other support; role of funders | Acknowledgements is included after the end of conclusion.  This work was supported by the Office of Global AIDS Coordinator and the U. S. Department of Health and Human Services, National Institutes of Health (NIH OAR and NIH OWAR) under Grant number 5R24TW008863. The contents of this publication are solely the responsibility of the authors and do not necessarily represent the official views of the government.  The funders were not involved in the: design of the study, data collection, statistical analysis of data, interpretation of the results, drafting of the manuscript and decision to submit the manuscript for publication. |
