# Supplementary material for: The performance of pre-delivery serum concentrations of angiogenic factors in predicting postpartum antihypertensive drug therapy following abdominal delivery in severe preeclampsia and normotensive pregnancy
Source: PLoS One. 2019 Apr 25;14(4):e0215807. doi: 10.1371/journal.pone.0215807 (PMC6485032; doi:10.1371/journal.pone.0215807)
Supplement: S2 Table — (DOCX) [file pone.0215807.s002.docx]

S2 Table. Statistically significant difference in the pre-delivery serum concentration of angiogenic factors between normotensive pregnancy and preeclampsia with severe features

| Gestational age category (and their frequency, n) | Angiogenic factor | *p*-value |
| --- | --- | --- |
| 34 – 36 weeks (n = 17) | sFlt-1 | <0.001 |
|  | PIGF | <0.001 |
|  | sFlt-1/PIGF ratio | <0.001 |
| 37 – 40 weeks  (n = 90) | sFlt-1 | <0.001 |
|  | PIGF | <0.001 |
|  | sFlt-1/PIGF ratio | <0.001 |
| 41 – 42 weeks  (n = 16) | sFlt-1 | 0.025 |
|  | PIGF | 0.014 |
|  | sFlt-1/PIGF ratio | 0.007 |

There was no normotensive participant delivered before 34 weeks of gestation to be compared with participants with preeclampsia with severe features. Abbreviations: PIGF, placental growth factor; sFlt-1, soluble fms-like tyrosine kinase-1.
